# Proximal humerus fractures in the pediatric population: a systematic review

**DOI:** 10.1007/s11832-011-0328-4

**Published:** 2011-03-11

**Authors:** Sohrab Pahlavan, Keith D. Baldwin, Nirav K. Pandya, Surena Namdari, Harish Hosalkar

**Affiliations:** 1 University of California, San Diego, San Diego, CA USA; 2 Children’s Hospital of Philadelphia, Philadelphia, PA USA; 3 Rady Children’s Hospital, University of California, San Diego, 3030 Children’s Way, Suite 410, San Diego, CA 92123 USA; 4 Hospital of the University of Pennsylvania, Philadelphia, PA USA

**Keywords:** Proximal humerus fracture, Children, Operative and non-operative

## Abstract

**Purpose:**

Proximal humerus fractures and epiphyseal separations in skeletally immature children and adolescents are traditionally treated non-operatively. Recently, authors have described the operative fixation of these injuries, particularly in older children and adolescents with displaced fractures. We performed a systematic review of the literature to identify operative indications for proximal humerus fractures in children and to compare the results by age, displacement, and treatment modality.

**Methods:**

A systematic review of the literature from January 1960 to April 2010 was performed. All studies with patients under the age of 18 years who were treated for a proximal humerus fracture either operatively or non-operatively were included.

**Results:**

The available literature is largely composed of uncontrolled case series (Level IV). According to findings, the literature shows that asymptomatic union is the rule in proximal humerus fractures in children and adolescents. Poorer outcomes were noted in operatively treated patients, patients with more displaced fractures, and older patients.

**Conclusions:**

The currently available literature supports a non-operative treatment approach, particularly in younger children with more growth remaining. Older patients (>13 years) with more widely displaced fractures may benefit from anatomic reduction with stabilization, though the data in the literature at this point is too weak to strongly recommend this approach. Further analysis with a more rigorous scientific method is necessary to evaluate the optimum treatment modality in this subgroup.

## Introduction

The treatment of pediatric proximal humerus fractures is rarely debated. The traditional teaching is that non-operative treatment is expected to give satisfactory results with return to full function and complete anatomic remodeling. Many studies have touted the unparalleled remodeling capacity of the proximal humerus in the skeletally immature population [1–4]. This is primarily a result of the fact that approximately 80% of the longitudinal growth of the humerus comes from the proximal humeral physis [4]. This unique biology forms the underpinnings of the historically wide acceptance of non-operative treatment regardless of the degree of displacement, angulation, rotation, or translation [1, 4–7].

In 1965, Charles Neer, in his classic paper, declared that, regardless of the degree and severity of displacement, open treatment of proximal humerus fractures in children is rarely justified [4]. He made these recommendations in spite of the fact that the majority of patients in his grade IV (displacement greater than 2/3rds of the humeral shaft) group had persistent deformity (described as “anterior bowing”) and many had notable arm shortening compared to the opposite side.

Since that time, several other studies published have attempted to address the reasons for potential malunion in many of these cases. These studies have alluded to the interposition of the periosteum or the long head of the biceps tendon within the fracture site as factors that block satisfactory restoration of alignment and fracture reduction with closed techniques [4, 7–12]. Many studies have described the limitation in range of motion and persistent pain in these displaced fractures treated both operatively and non-operatively [1, 6, 7, 9, 10, 13, 14]. It remains unclear whether this decreased motion is the result of soft tissue interposition, altered mechanics of the rotator cuff, or some other unrecognized factor [1, 15]. Although some studies blame surgical treatment for poor outcomes, the selection bias of these level IV case series remains in that operative treatment is more likely to be undertaken in more displaced fractures with potentially greater soft tissue and/or bony injury [3, 5, 6, 10]. Other studies have recognized age as an important factor in the treatment of these fractures [3, 9, 10–12]. Older adolescents with less remodeling capacity are thought to have worse outcomes with non-operative treatment for malreduced fractures than younger children with greater opportunity for growth and remodeling [3, 9–11].

We, therefore, performed a systematic review of the literature to answer the following questions. What are the outcomes of the treatment of proximal humerus fractures in the pediatric population? Are there any subgroups of patients that may benefit from operative treatment (for example, more displaced, older adolescents)? What are the reported complications of operative and non-operative treatment of these injuries?

## Materials and methods

**Fig. 1:**
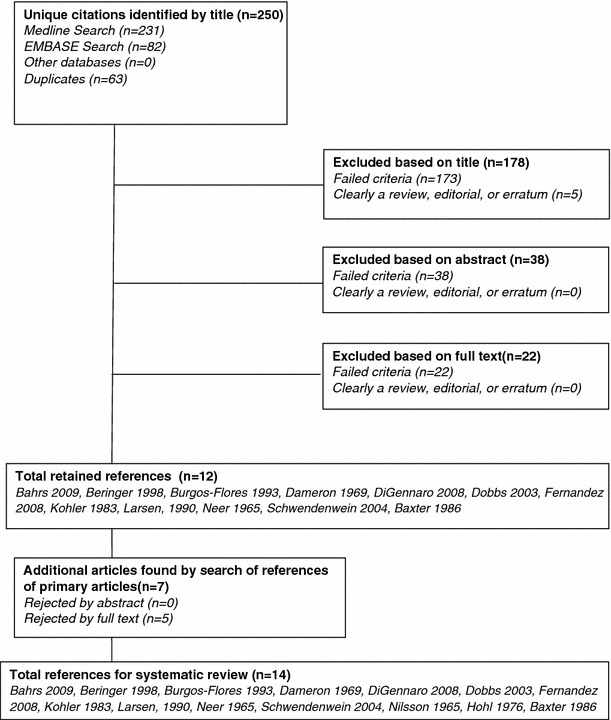
Process of article selection

We searched the Medline and EMBASE databases from January 1960 to April 2010 for articles using the following search terms: “proximal humerus” and fracture(s). We used limits of “English language” and “all children 0–18”. Reference lists from the articles retrieved were further examined to identify any additional studies of interest. Studies were included in this systematic review if they met the following criteria: (1) they were available in English, (2) they had a level I–IV study design by the Journal of Bone and Joint Surgery criteria (since the majority of studies in the clinical orthopedic literature are retrospective studies of level III–IV evidence, our goal was to be inclusive), (3) patients in the study had a proximal humerus fracture or epiphyseal separation, (4) each study had at least 15 proximal humerus fractures in children, (5) all patients included in the study were younger than 18 years of age, or those younger than 18 years could be individually analyzed, (6) there was a distinct treatment and/or outcome (i.e., not just a technique article), (7) studies were published in or after 1960. The search algorithm and results by phase of the search are detailed in Fig. 1. Ultimately, 14 articles met our inclusion criteria when the search was finalized.

Two authors (S.P., K.D.B.) reviewed articles by title; three (S.P., K.D.B., N.K.P.) reviewed the articles by abstract; four reviewed full text for inclusion and content (S.P., K.D.B., S.N., N.K.P.). The overall agreement on which articles should be reviewed by full text was 79% (free marginal kappa 0.583, moderate agreement) [16]. In terms of the reasons for exclusion, 76 articles were excluded because they did not fulfil the inclusion criteria of having at least 15 pediatric subjects undergoing treatment of proximal humerus fracture, including case reports and technical articles where data regarding proximal humeral fractures could not be extracted. One hundred and eighteen articles were excluded because they focused on injuries that were not related to the proximal humerus or dealt with chronic injuries. Thirty articles were excluded because they were epidemiologic, review articles or other studies without a primary treatment arm. Fourteen articles were excluded because they were primarily radiographic articles with no focus on the treatment of proximal humerus fractures. If there was disagreement on whether the full text of an article should be reviewed, the article was included for review. Ultimately, 34 articles were reviewed by full text; of these articles, 12 were deemed appropriate for the review given our inclusion criteria. Of the remaining 12 articles, a review of the bibliographies was done by the reviewing authors, who selected articles to be further reviewed for inclusion. Seven additional articles were identified using this method and the text of these articles were further reviewed. One of these studies was rejected because it did not have a sufficient number of pediatric patients [17]. Three papers were rejected because the data on proximal humeral fractures could not be isolated from the rest of the data presented [18–20]. One paper was rejected because it was a review article [21]. Two articles met the inclusion criteria and were included in the review [2, 14]. After reviewing these articles, we were left with a total of 14 articles (Fig. 1). In the final step, there was 100% agreement on the articles which should be included in the final investigation.

Data from these 14 studies was subsequently extracted and reviewed by all authors (S.P., K.D.B., N.K.P., S.N., H.H.). This data included: outcomes of operative versus non-operative patients, outcomes of severely (Neer-Horowitz IV) versus less severely (Neer-Horowitz I–III) displaced fractures, and older (13 years of age or greater) patients versus younger patients. The parent studies incorporated a total of 765 proximal humerus fractures in pediatric patients; 569 had adequate follow up for outcomes. The patient ages ranged from under a year old to 18 years of age. Average ages ranged from just under 10 years of age to 13.5 years of age. Sixty-one percent of the patients were male. Twenty-nine patients were operatively treated (closed reduction percutaneous pinning; open reduction, internal fixation; or open reduction only), and the remainder were non-operatively managed (sling, sling and swathe, traction, splint, or hanging cast). The full demographic data are shown in Table 1.

**Table 1:** Study demographics

Study	Year	Journal	Level	No. of patients (with follow up)	Average age, years (range)	Average follow up, years (range)	M/F	Operative/non-operative
Neer and Horowitz [4]	1965	Clin Orthop Relat Res	IV	89 (62)	(1–17)	(2–11)	67/22	22/67
Nilsson and Svartholm [14]	1965	Acta Chir Scand	IV	44 (44)	9.8* (4–16)	7.8 (1–15)	28/16	6/38
Dameron and Reibel [1]	1969	J Bone Joint Surg Am	IV	69 (46)	(0.8–17)	7 (0–17)	42/27	3/63
Hohl [2]	1976	Orthop Clin North Am	IV	15	12 (9–15)	n/a	8/7	1/14
Kohler and Trillaud [3]	1983	J Pediatr Orthop	IV	136 (52)	(1–16)	5 (1–18)	82/54	36/100
Baxter and Wiley [5]	1986	J Bone Joint Surg Br	IV	57 (30)	n/a (8.9–15.1)	2.7 (2–8)	31/26	7/50
Larsen et al. [13]	1990	Acta Orthop Scand	IV	72 (64)	11 (0–15)	9 (8–11)	38/34	0/72
Burgos-Flores et al. [9]	1993	Int Orthop	IV	22	13 (7–19)	6.8 (2–11.4)	15/7	22/0
Beringer et al. [6]	1998	J Pediatr Orthop	IV	48 (21)	13.5	9	n/a	9/39
Dobbs et al. [11]	2003	J Pediatr Orthop	IV	28	(5–16)**	4 (2–14)	24/4	25/3
Schwendenwein et al. [7]	2004	Eur J Pediatr Surg	IV	16	10.6 (4–15)	2 (0.7–6)	9/7	15/1
Di Gennaro et al. [10]	2008	Chir Organi Mov	IV	91	10.7	Non-operative 0.6 (0.1–15) Operative 2.9 (0.1–14.5)	52/39	9/82
Fernandez et al. [12]	2008	Injury	IV	35	12.7	2.2 (0.5–4.8)	15/20	35/0
Bahrs et al. [8]	2009	J Pediatr Orthop	IV	43	Non-operative 12.4 (6–16) Operative 14 (6–18)	3.3 (1–10)	29/14	33/10

Operative treatments include CRPP, ORIF, ORPP, or OR with immobilization

*Median age

**No mean age specified

No systematic assessment of study quality was performed, since all studies were level IV case series and, hence, did not routinely use rigorous scientific technique. No study reviewed was prospective. No study reviewed blinded the assessment of outcome to treatment modality, amount of displacement, or age of the patient. No study reviewed used statistical adjustment for multiple factors which could be responsible for patient outcome. Many studies had incomplete follow up; in fact, some studies had less than 50% of patients with follow up [1, 3, 5, 6]. Follow up clinical and radiographic assessment was either not described or not standardized in all studies. The study populations were defined, but outcomes for all subgroups were not routinely available in all studies. Few studies explicitly stated their operative indications.

## Results

### Overall outcomes

The overall outcomes were reported to be excellent. Follow up ranged from 0.6 to 9 years. No non-unions were reported. Malunions were scarce, and the malunions that were reported were well tolerated at the last reported follow up [1, 3, 4, 13, 14]. The vast majority of patients in the studies examined returned to unimpeded function without major complications. There were, however, reports of post-injury stiffness or loss of motion [1, 10, 13, 14], pain [1, 7, 10, 13, 14], weakness [1, 14], nerve symptoms [14], growth plate injury [3], and functional limitations [1, 12]. Interestingly, earlier papers (1965–1990) uniformly recommend non-operative therapy [1, 2–4, 13, 14]. The one exception to this rule was Baxter and Wiley, who recommended operative treatment only in the setting of vascular injury or skin tenting [5]. Studies in the 1990s were mixed. Beringer et al. (1998) recommended non-operative treatment almost exclusively, unless there was articular involvement or another compelling reason to operate [6]. This is in contrast to Burgos-Flores et al. (1993), who recommended surgical treatment in children over 13 years of age with displaced fractures [9]. Articles published in the 2000s recommended surgery for fractures with residual displacement [7, 8, 10–12], particularly for older children [10–12]. Seven of the reviewed studies (50%) noted between one and seven patients with a biceps tendon which was interposed in the fracture site, blocking anatomic fracture reduction [4, 7–12]. In fact, of the 223 operatively treated patients, 21 were found to have biceps interposition in the fracture site (9.4%).

### Outcomes by displacement

Four studies compared outcomes by the amount of displacement [4, 8, 9, 13]. We compared Neer-Horowitz type IV fractures (displaced greater than 2/3rds of the humeral shaft) with fractures displaced less than this (Neer-Horowitz I, II, and III) [4]: 22% of patients with type IV fractures had pain at final follow up, compared to 7% of less displaced fractures; 39.5% of type IV fractures had some shortening at final follow up, compared to 13.3% of less displaced fractures; 12% of patients with grade IV fractures had restriction of motion at final follow up, compared to 6% of patients with less displaced fractures; 10% of patients with grade IV fractures had angulation >20° at final follow up. No patient with less displacement exhibited this degree of angulation at follow up (Table 2).

**Table 2:** Fracture outcomes by displacement

Study	Pain at follow up, grade I–III	Pain at follow up, grade IV	Shortening at follow up, grade I–III	Shortening at follow up, grade IV	Restriction of motion at follow up, grade I–III	Restriction of motion at follow up, grade IV	Angulation >20° at follow up, grade I–III	Angulation >20° at follow up, grade IV
Neer and Horowitz [4]	n/a	n/a	6/71^a^	5/13^b^	n/a	n/a	n/a	n/a
Larsen et al. [13]	3/42	4/10	0/42	0/10	1/42	1/10	0/42	1/10
Burgos-Flores et al. [9]*	1/14	0/8	6/14^c^	4/8^d^	4/14	2/8	n/a	n/a
Baxter and Wiley [5]	n/a	n/a	8/23^e^	6/7^f^	0/23	0/7	n/a	n/a
Total	4/56	4/18	20/150	15/38	5/79	3/25	0/42	1/10

^a^Four with >2 cm shortening

^b^Four with >2 cm shortening

^c^Three with 2 or greater cm

^d^One with >2 cm

^e^One with 1.5–2 cm; three with 1–1.5 cm; four with 0.5–1 cm shortening

^f^Two with 1.5–2 cm; three with 1–1.5 cm; one with 0.5–1 cm shortening

*All were either CRPP or ORIF

### Outcomes by treatment

Six studies compared outcomes by treatment [1, 2, 5, 8, 10, 14]. Bahrs et al. reported Constant scores, noting a superior mean Constant score in non-operatively treated patients [8]. The other studies used other outcomes such as pain, shortening, restriction of motion, and angulation. Three studies had pain at follow up as an outcome that was stratified by treatment [1, 10, 14]. Of the patients analyzed in these studies, 22% of the operative patients had pain at follow up, compared to 7% of the non-operative patients. Two studies reported shortening at final follow up by treatment [2, 4]. No operative patient had shortening in these studies. Four percent of non-operative patients had some degree of shortening. Three studies reported restriction of motion at final follow up stratified by treatment [1, 10, 14]. These studies reported that 28% of their operative patients had restriction in motion at final follow up, compared to 6% of the non-operatively treated patients.

### Outcomes by age

Only three studies stratified outcomes by age [5, 8, 9]. In the series by Burgos-Flores et al., 25% of their patients under 13 years of age had shortening compared to 57% of their patients over 13 years of age [9]. Twenty-five percent of their patients under 13 years of age had restriction in range of motion at final follow up compared to 29% of their patients over 13 years of age. All were treated operatively in this series. Baxter and Wiley reported humeral shortening according to age and concluded that age at the time of fracture did not influence the ultimate amount of shortening [5]. Bahrs et al. noted Constant scores in their patients. Their non-operative patients all had 100 Constant score post-treatment. The average Constant score for operatively treated patients was 89 in the 10 years and younger group and 95 in the 10 years and older group [8]. The authors of this series report no shortening or restriction of motion [8].

## Discussion

Proximal humerus fractures comprise 0.45% of all fractures in children and 4–7% of all epiphyseal fractures [4, 22, 23]. The proximal humeral physis contributes to approximately 80% of humeral length [1, 4]. Traditionally, proximal humerus fractures in skeletally immature patients have been treated non-operatively due to the tremendous potential for remodeling and the wide functional arc of motion of the shoulder. As a result, even significantly angulated and displaced fractures have achieved union in positions that have allowed for normal or near-normal functional outcome. In children up to 10 years of age, axial malalignment of the proximal humerus of as much as 60° in varus, anteversion, or retroversion can be corrected by remodeling; however, beyond 10 years of age, the remodeling potential is not as high and correction can be expected only with axial deformities of up to 20–30° [12]. It is important to note that the majority of previously published outcome studies evaluating displaced proximal humeral epiphyseal fractures included few patients that were 15 years or older [11]. Additionally, there is some concern that early studies that did include adolescent patients found worse outcomes in severe fractures treated non-operatively [1, 6, 9]. As early as 1969, Dameron and Reibel evaluated 46 patients with proximal humeral physeal fractures and noted poor outcomes in patients aged 14 years or older who lost fracture reduction during the treatment period [1].

While the only absolute indications for the fixation of a proximal humerus fracture in a skeletally immature patient include open fracture or neurovascular injury, relative indications have become increasingly widened with the emergence of new data. Much of the published data regarding the non-operative management of proximal humerus fractures in skeletally immature patients include a large number of non-displaced or minimally displaced fractures and small numbers of older children or adolescents [24]. Additionally, the published literature is inadequate in the stratification of proximal humerus fractures by age and displacement. In later years, authors have expressed a willingness, and even a desire, to intervene surgically in older patients with more displaced fractures. Consequently, operative indications have expanded in this group, with many authors advocating the operative treatment of widely displaced proximal humerus fractures in adolescents [6]. Though traditionally treated in a non-operative fashion due to the expected remodeling potential of the proximal humerus, high-demand adolescent patients may be undertreated. Additionally, it is unclear what the long-term effects of subtle to moderate malalignment has on glenohumeral mechanics and eventual secondary arthritis of the glenohumeral articulation. Almost 10% of severely displaced fractures may have biceps tendon interposition, although the long-term effects of this neglect is not known.

Our systematic review demonstrates a paradigm shift in the age-based treatment philosophy, as earlier reports focused on non-operative management, while more recent investigations have widened the relative indications for surgery, particularly in older children. Presumably, this widening of indications is a result of the poorer outcomes noted in older patients [5, 8, 9], particularly those with more displacement [4, 8, 9, 13]. Proximal humeral fractures in children need to be stratified and treated on an individual basis. Our recommendation based on this systematic review would be to divide them into three groups: (a) <10 years, (b) 10–13 years, and (c) >13 years. Children <10 years of age can be nearly universally treated in a non-operative fashion successfully due to the expected high remodeling potential of the proximal humerus. Those above 13 years of age with limited remodeling capacity should certainly be offered the choice of appropriate alignment and fixation and be allowed to come to an informed decision, provided the displacement of their fracture warrants it. The interim group should be treated on a case-to-case basis, including their gender, true bone age, and biological capacity to remodel.

Absolute criteria for the amount of displacement and angulation as an indication for surgical fixation have not been clearly established by the published literature. Burgos-Flores et al. described their operative indications as a patient with a fracture over 30% angulated or 50% displaced [9]. They temper their recommendations at the end of their paper, however, by adding that this degree of angulation should be accepted in children under 13 years of age and treated aggressively in children over 13 years of age [9]. Our literature review revealed only three studies that reported outcomes based on age cutoff. Burgos-Flores et al. noted that 13 years of age was an appropriate cutoff, whereas Bahrs et al. reported Constant scores above and below 10 years of age [8, 9].

Traditionally, operative management has included obtaining a closed or open reduction, followed by stabilization and fixation with multiple options, including wires, cannulated screws, retrograde elastic stable intramedullary nailing (ESIN), or a plate. Several studies have demonstrated excellent results with all of these surgical techniques and suggest that the anatomic reduction of severely displaced proximal humerus fractures is justified, especially in patients over 13 years of age [6, 9, 7, 12, 25]. Burgos-Flores et al. noted excellent results in 22 patients with Neer grade III and IV proximal humeral epiphyseal fractures treated with closed or open reduction and wire fixation at a mean of 6.8 years of follow up. They noted that, since there is a greater occurrence of residual deformity and limitation of motion in older patients, a more aggressive approach to correct the initial displacement and angulation is warranted in those over the age of 13 years. Rajan et al. examined 14 patients (10–15 years of age) with severely displaced proximal humerus physeal fractures who underwent reduction and stabilization with ESIN at a mean of 30 months follow up, noting excellent functional outcomes and 100% union [24]. Fernandez et al. reported on 35 children (mean age 12.7 years) who underwent ESIN of proximal humerus fractures at 26 months follow up and noted improved functional outcomes and return to sports in all patients [12].

The major weakness of this study is that, because it is a systematic review of observational studies, it contains all of the biases inherent to the studies which were used to comprise it. Most notably, only a small proportion of studies actually delineated their indications for surgical intervention [2, 5]. Although age, displacement, and treatment type are clearly important factors influencing the outcome from reviewing the studies, only one study stratified by these factors [8], while one stratified by age and treatment [5] and one stratified by age and displacement [9]. As such, it is nearly impossible to determine when operative treatment is indicated, though studies in the most recent decade have been more aggressive in recommending surgery for these injuries in older children [7–12]. Although the age cutoffs of <10, 10–13, and >13 years are good numbers to practically help clinicians with decision-making, biologically, it may be difficult to parse out because skeletal maturity does not necessarily match chronologic age perfectly. In the same vein, a gold standard surgical technique has not been established for operative treatment. Unfortunately, the available literature is not sufficient to either justify or refute this practice, as it lacks stratification or statistical adjustment necessary to note differences in this specific population. In actuality, it lacks even the requisite stratification necessary to aggregate data for a meta-analysis. Clearly, however, based on the evolution of the literature, with advancing training and evolving techniques, there is a desire on the part of the clinical community to identify appropriate operative indications in this population in order to improve outcomes in older patients with more displaced fractures.

Prospective or more rigorously performed retrospective studies are necessary to define the specific older children and adolescent candidates that would benefit from anatomic reduction with stabilization using modern techniques with closed or open means. The reader should be cautioned, however, before this recommendation can be strongly made; better studies of the outcomes of these patients are necessary in order to answer the following questions: what are the long-term outcomes of patients older than 10–13 years treated operatively and non-operatively with severely displaced fractures? What is the long-term fate of the biceps tendon and glenohumeral articulation? What are the patient-centered outcomes at 10, 20, and 30 years from the initial injury? These questions remain unanswered by the literature, and, as such, by this systematic review.
